# Anlotinib combined with tislelizumab in the treatment of primary small cell neuroendocrine carcinoma of the prostate: a case report and literature review

**DOI:** 10.3389/fimmu.2024.1510069

**Published:** 2024-12-23

**Authors:** Xin Fei, Zhong Zheng, Zhen-ya Zhao, Da-wei Ren, Su-ying Wang, Shi-jie Ye, Lin-chun Liang, Da Li, Xiao-long Jia, Qi Ma

**Affiliations:** ^1^ Department of Health Science Center, Ningbo University, Ningbo, China; ^2^ Department of Urology, The First Affiliated Hospital of Ningbo University, Ningbo, China; ^3^ Department of Imaging, The First Affiliated Hospital of Ningbo University, Ningbo, China; ^4^ Department of Pathology, Ningbo Clinicopathological Diagnosis Center, Ningbo, China; ^5^ Department of Medical Oncology, Mingzhou Hospital, Zhejiang University, Ningbo, China; ^6^ Department of Medical Oncology, Sir Run Run Shaw Hospital Affiliated to Zhejiang University Medical School, Hangzhou, China; ^7^ Yi-Huan Genitourinary Cancer Group, The First Affiliated Hospital of Ningbo University, Ningbo, China; ^8^ Comprehensive Genitourinary Cancer Center, The First Affiliated Hospital of Ningbo University, Ningbo, China

**Keywords:** small cell neuroendocrine carcinoma, NEPC, case report, anlotinib, tislelizumab, prostate cancer

## Abstract

Primary small cell neuroendocrine carcinoma of the prostate is extremely rare, highly aggressive, and has a very poor prognosis, with an overall survival typically not exceeding one year. Standard treatment is generally based on the regimen for small cell lung cancer (SCLC), with guidelines recommending etoposide combined with cisplatin (EP regimen) as the first-line treatment. However, their therapeutic effects are limited. For primary small cell neuroendocrine carcinoma of the prostate that has failed the EP regimen treatment, there is currently a lack of relevant treatment methods. Here, we report a case of small cell neuroendocrine carcinoma of the prostate with multiple metastases, whose disease rapidly progressed despite receiving EP and second-line systemic chemotherapy. The patient was then administered a combination of anlotinib and tislelizumab. After treatment, the patient’s symptoms were controlled, tumor marker levels decreased, and imaging showed significant improvement. The patient had a progression-free survival time of more than 22 months and continued to receive treatment. This is the first report of the use of anlotinib combined with tislelizumab for the treatment of primary small cell neuroendocrine carcinoma of the prostate, providing a new therapeutic option for patients with this disease.

## 1 Introduction

Neuroendocrine prostate cancer (NEPC) is characterized by low or absence of androgen receptor (AR) expression and an increased neuroendocrine phenotype, with an overall survival typically not exceeding one year. Clinically, NEPC can be classified into primary (*de novo* NEPC or dn-NEPC) and treatment-induced (t-NEPC) types. The former refers to neuroendocrine tumors present at the initial diagnosis, accounting for only 2% of all cases (1), and is usually more aggressive than t-NEPC. The latter refers to neuroendocrine tumors induced by androgen deprivation therapy (ADT), with an incidence of up to 17% in castration-resistant prostate cancer (CRPC) (1), making it a highly aggressive histological subtype of CRPC (2). Primary NEPC can be divided into several histological types including common prostate cancer with neuroendocrine differentiation, adenocarcinoma with Paneth cell neuroendocrine differentiation, and carcinoid, small cell, and large cell neuroendocrine carcinoma (3) (**Table 1**). Among them, pure small cell neuroendocrine carcinoma is the most aggressive type of NEPC, with the worst prognosis, and the overall survival (OS) is generally within one year (4–6).

**Table 1:** Histological subtypes of neuroendocrine prostate cancer.

Histological Subtype	Morphological Features	Immunohistochemistry	Proliferation Index
Small Cell Neuroendocrine Carcinoma	Poorly defined cell boundaries, high nuclear-cytoplasmic ratio, high mitotic rate, and numerous apoptotic bodies. Electron microscopy shows neuroendocrine granules.	Ki-67 (++), CgA (+), SPY (+), p63 (+), CK (+)	+++
Large Cell Carcinoma	Large tumor cell nests with palisading arrangement around the nests, often accompanied by geographic necrosis; cells have non-Small Cell Neuroendocrine Carcinoma morphology, large nuclei, abundant cytoplasm, amphophilic, prominent nucleoli, coarse chromatin, and easily visible mitotic figures.	CD56 (++), CD57 (++), CgA (++), SPY (++), P504S (++); Ki-67 (+)	++
Carcinoid	Characterized by nest-like structures and uniform nuclei, cytoplasm shows “salt and pepper” chromatin or scattered Paneth cell-like eosinophilic granules; may show immunophenotypic and/or ultrastructural features of neuroendocrine differentiation.	PSA (+)	–
Adenocarcinoma with Paneth Cell-like Neuroendocrine Differentiation	Marked increase in eosinophilic cells, electron microscopy shows neuroendocrine granules.	CgA (+), Ki-67 positive index usually low, PSA (−)	Variable
Typical Adenocarcinoma with Neuroendocrine Differentiation	Typical acinar or ductal prostate adenocarcinoma with eosinophilic granules and nuclei with salt and pepper chromatin.	–	–
Mixed Neuroendocrine Carcinoma/Adenocarcinoma	Overlapping morphology with varying proportions of small cells having neuroendocrine features (small nests, nuclear molding, fine chromatin) mixed with large cells showing adenocarcinoma phenotype.	–	++

CgA, Chromogranin A; SPY, Synaptophysin; CK, Cytokeratin; P504S, α-Methylacyl-CoA Racemase. Proliferation index indicated by Ki-67 expression. +/- refer to relative level of proliferation as assessed by Ki-67.

There is no standard treatment for primary NEPC, and treatment is often referred to as the small cell lung cancer (SCLC) protocol. Here, we report an elderly patient with primary small cell NEPC who experienced disease remission and achieved radiographic progression-free survival (rPFS) over 22 months after failure of platinum-based therapy and subsequently received anlotinib combined with tislelizumab immunotherapy. This is the first case reported in the literature of anlotinib combined with tislelizumab for the treatment of primary small cell NEPC. To our knowledge, this patient has the longest rPFS reported in the literature.

## 2 Case presentation

**Figure 1:**
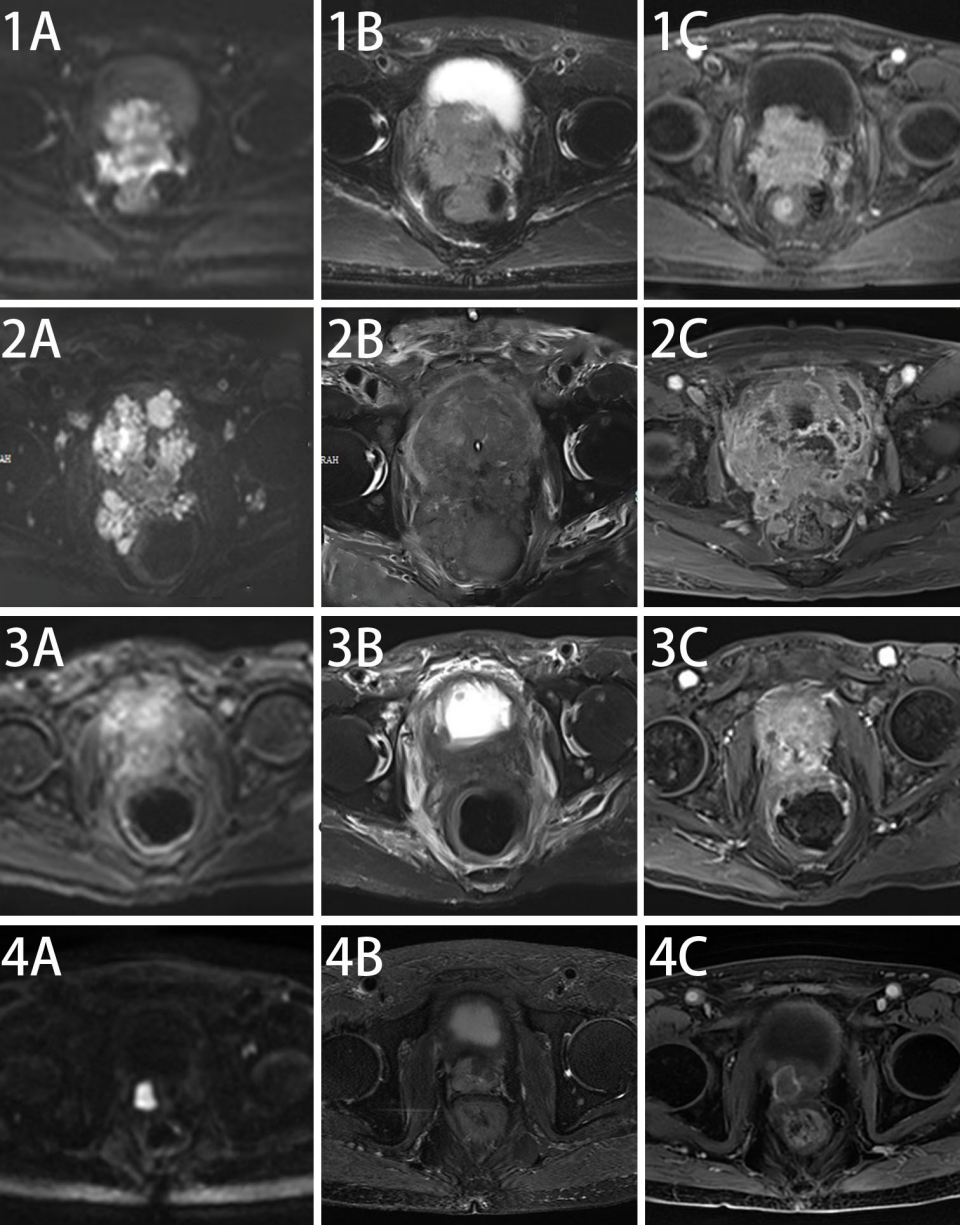
Changes in pelvic MRI images before and after treatment in this patient. Pelvic MRI Scan at diagnosis of this disease **(1A–C)**, after chemotherapy **(2A–C)**, after five cycles of anlotinib + tislelizumab treatment **(3A–C)** and maintenance treatment of anlotinib + tislelizumab **(4A–C)**.

The elderly patient presented with a history of urinary frequency and urgency for over 10 years, which had worsened over the past half month. Transrectal ultrasound in December 2021 indicated a prostate volume of 53 ml, with PSA levels of 1.71 ng/ml. A pelvic MRI [**Figures 1(1A–C)**] performed in January 2022 suggested the following: (1) malignant prostate lesion invading the bladder, seminal vesicles, and rectum; and (2) multiple enlarged pelvic lymph nodes.

**Figure 2:**
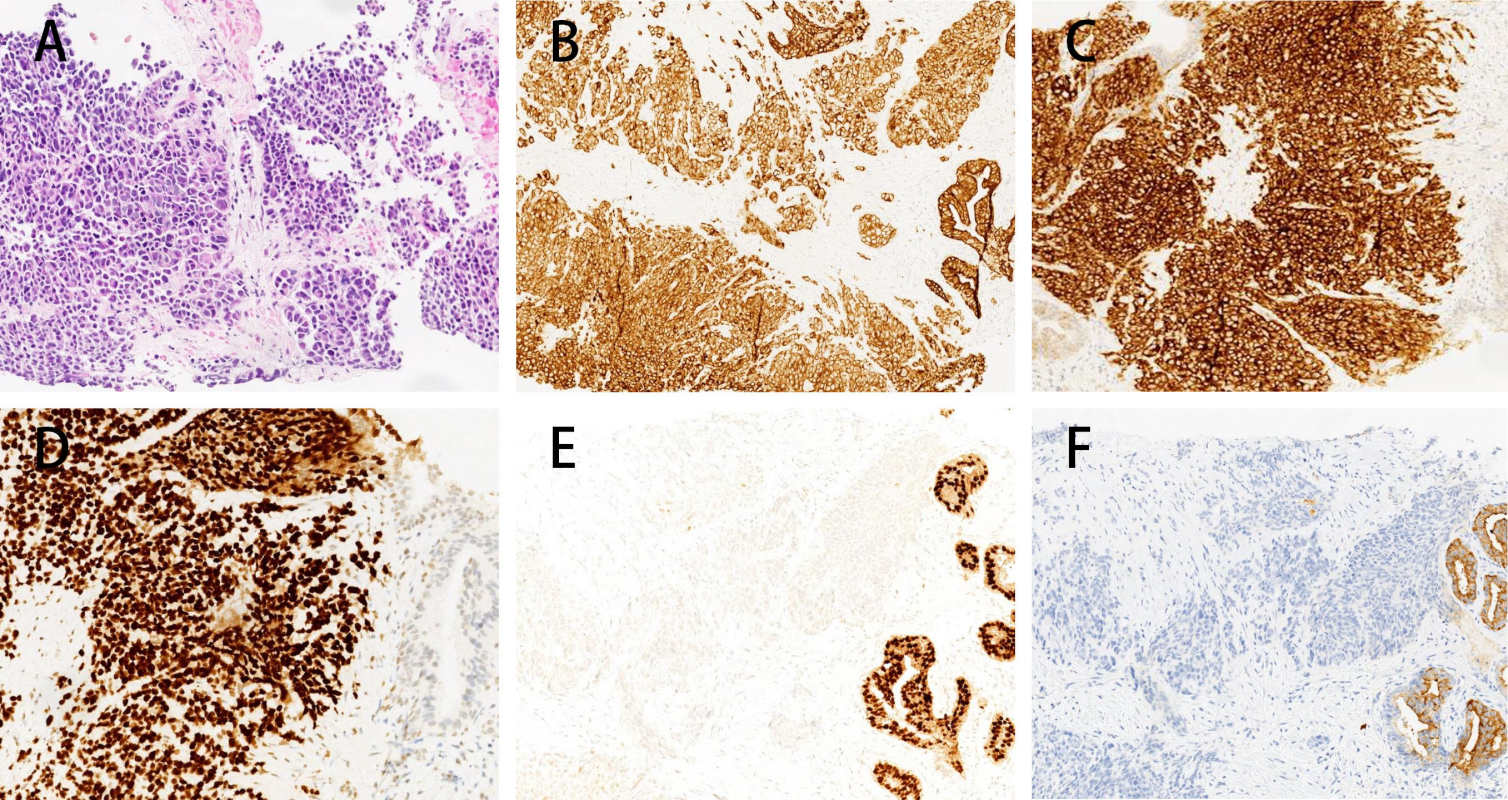
Immunohistochemistry of prostate biopsy specimens in this patient. Immunohistochemical analysis of the prostate biopsy specimen showing microscopic features of nests, cords, and diffuse growth of small round cells. The tumor cells exhibit a profile consistent with poorly differentiated neuroendocrine carcinoma (small cell carcinoma) **(A)**, characterized by positive pan-cytokeratin (CK) staining **(B)**, strong staining for Synaptophysin (+++) **(C)**, strong TTF-1 expression (+++) **(D)**, focal weak NKX3.1 staining **(E)** and PSA-negative expression in the tumor area and a small area with PSA positive expression in the normal prostate tissue **(F)**.

A prostate biopsy in January 2022, revealed “poorly differentiated neuroendocrine carcinoma (small cell neuroendocrine carcinoma).” Biopsy samples from various prostate regions revealed small round cells with nest-like, cord-like, and diffuse growth patterns. The immunohistochemical staining results were consistent with poorly differentiated neuroendocrine carcinoma (small cell neuroendocrine carcinoma). The results showed: PSA (−), PSAP (−), NKX3.1 (weakly in individual cells), Ki-67 (+ 90%), Syn (+++), CgA (+ scattered), CD56 (++), 34βE12 (+), P504S (±), AR (−) (**Figure 2**), PD-L1 (−) (**Supplementary Figure 1**).

**Figure 3:**
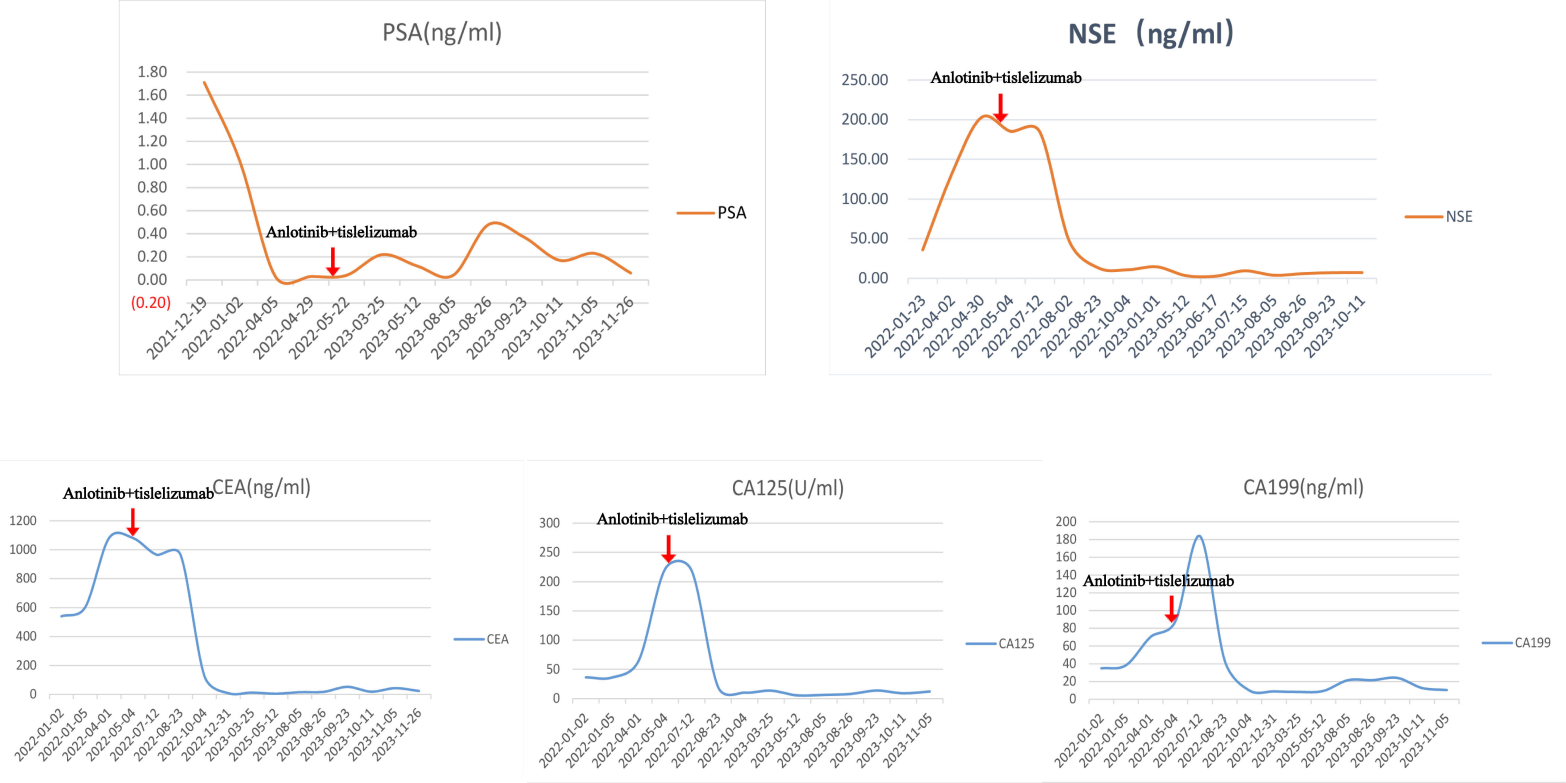
Patient’s PSA, NSE, and related tumor markers of this patient. The changes in PSA (prostate-specific antigen), NSE (neuron-specific enolase), and related tumor markers (CEA, CA125, CA199) before and after anlotinib and tislelizumab treatment in this patient.

The patient’s creatinine clearance rate was 46.65 ml/min, and PSA levels were low (**Figure 3**), while NSE and other tumor markers were elevated significantly (**Figure 3**), consistent with the pathological diagnosis. Based on pathology and imaging, he was diagnosed as “primary small cell neuroendocrine carcinoma of the prostate.” The patient had an ECOG score of 0, indicating good general condition.

To determine metastasis, a ^18^F-FDG PET-CT scan in January 2022, showed “prostate malignancy involving bilateral seminal vesicles, posterior bladder wall; multiple lymph node metastases around the prostate and along bilateral iliac vessels; liver metastasis in segment S3; bone metastasis in T10 and S1 vertebral body,” indicating multiple metastases including lymph nodes, liver, and bone of this highly aggressive tumor.

Currently, the NCCN Guidelines for the treatment of primary small cell NEPC are referred to as the NCCN Guidelines for SCLC. Considering the patient’s age and low creatinine clearance rate, chemotherapy with a decreased dose of the EP regimen (etoposide 300 mg + cisplatin 60 mg) was administered in February 2022 and March 2022. Despite two rounds of chemotherapy, NSE and related tumor markers continued to increase (**Figure 3**), and imaging indicated further progression. During treatment, the patient’s renal function quickly deteriorated. Abdominal CT scans showed hydronephrosis in both kidneys, and the tumor has invaded the bilateral ureteral orifices (**Supplementary Figure 2**. The details are provided in **Supplementary Videos 1**, **2**). Thus, bilateral percutaneous nephrostomy was performed to rapidly relieve obstruction and improve renal function. The patient also experienced severe gastrointestinal side effects during EP chemotherapy. Given the tumor markers, imaging progression, symptomatic progression, and treatment side effects, first-line EP chemotherapy was deemed to fail. The patient’s ECOG score was 2.

After a multidisciplinary team (MDT) discussion and reference to the NCCN guidelines, the patient received second-line chemotherapy with “irinotecan 100 mg + carboplatin 140 mg” in April 2022. Following chemotherapy, NSE and related tumor markers continued to increase (**Figure 3**), and imaging indicated further progression [**Figures 1(2A–C)**]. The patient also experienced significant bone marrow suppression and fatigue during the chemotherapy. Given the elevation of tumor markers, imaging progression, symptomatic progression, and treatment side effects, second-line treatment was also deemed to fail. The patient’s ECOG score was 3.

Since there is no standard treatment for primary small cell NEPC, and the tumor is rapidly progressing, another MDT discussion was held to provide a new treatment strategy for this patient. After thorough discussion and informed consent from the patient and his family, innovative therapy was administered to this patient. In May 2022, the patient received combined targeted immunotherapy with “anlotinib 8 mg/day for 14 days + tislelizumab 200 mg/day per 21 days,” administered every three weeks. After the 5th cycle of combined targeted and immunotherapy, on August 2022, pelvic MRI [**Figures 1(3A–C)**] and tumor markers, including NSE (**Figure 3**), showed tumor shrinkage and a significant decline in tumor markers, proving the therapy’s effectiveness.

A follow-up ^18^F-FDG PET-CT on November 2022, indicated: “Compared to January 2022 PET-CT: the prostate is significantly smaller, FDG metabolism significantly reduced; Scattered small lymph nodes around the prostate and bilateral iliac vessels, no increased FDG metabolism; liver metastasis in segment S3, no abnormal increase in FDG metabolism; Low metabolic osteoblastic metastases in T10 and S1 vertebral body.” The patient’s ECOG score improved to 1.

However, in November and December 2022, the patient experienced two episodes of severe COVID-19 pneumonia. He had to pause the anti-tumor treatment. After recovering from COVID-19, owing to the cessation of anti-tumor therapy, the patient developed severe back pain, particularly at night and after carrying heavy loads. Upon re-evaluation in March 2023, MDT discussions led to the resumption of “anlotinib + tislelizumab” treatment in March 2023.

Unfortunately, in November 2023, the patient experienced stroke. MDT discussions considered that the possibility of the combined treatment of anlotinib and tislelizumab was low. After recovery from stroke, he continued anti-tumor treatment, although his ECOG score was 3.

The patient survived for over two years, receiving 25 cycles of treatment, maintaining good general condition, and no significant side effects were observed during treatment. On 16 March 2024, pelvic MRI showed no significant progression [**Figures 1(4A–C)**].

Considering the patient’s age and the impact of two bouts of COVID-19 infection and stroke during anti-tumor therapy, the overall survival may be affected. However, after the diagnosis of primary small cell NEPC with multiple lymph node, liver, and bone metastases, the patient has survived for more than two years, which is, to our knowledge, the longest survival reported in the literature to date.

## 3 Discussion

Primary small cell neuroendocrine carcinoma of the prostate is a poorly differentiated neuroendocrine prostate cancer (NEPC) that is extremely rare (<1%), highly aggressive, and has a very poor prognosis, with an overall survival typically not exceeding one year (4, 5). It often presents with symptoms related to local infiltration or metastatic disease, including bowel or bladder invasion; hydronephrosis; metastatic disease to visceral organs such as the liver, lungs, and central nervous system; and primarily osteolytic bone metastases (7). Currently, the treatment of NEPC often references the treatment strategy for small cell lung cancer (SCLC). According to the 2022 NCCN SCLC guidelines, the first choice is chemotherapy with a platinum-based drug combined with etoposide (EP regimen) is recommended for patients with limited-stage disease (8). For patients with extensive-stage disease, the preferred treatment is a platinum-based drug combined with etoposide and PD-L1 inhibitors, such as atezolizumab or durvalumab (9). However, even with the EP regimen combined with PD-L1 inhibitors, primary NEPC still exhibits extremely low survival rates and poor prognoses. Wee et al. analyzed seven NEPC patients who received a combination treatment of carboplatin, etoposide, and atezolizumab (two of whom had newly diagnosed small cell neuroendocrine carcinoma of the prostate) and found that the median follow-up time was 6.5 months (range: 1.5–15.1), with a median progression-free survival (mPFS) of 3.4 months and a median overall survival (mOS) of only 8.4 months (with the longest patient OS being 15.1 months) (5). Therefore, the treatment of primary NEPC faces challenges of both rarity and poor efficacy.

Here, we report an innovative treatment approach for primary small cell neuroendocrine carcinoma of the prostate in an elderly patient. The patient, with advanced age, had a low creatinine clearance rate and an ECOG performance status score of 0. After the pathological diagnosis of primary small cell NEPC, imaging revealed multiple lymph node, liver, and bone metastases. According to the 2022 NCCN SCLC guidelines, platinum-based drugs should be combined with etoposide and PD-L1 inhibitors such as atezolizumab or durvalumab. Since atezolizumab/durvalumab was not yet approved in China for this indication in 2022, the patient received an EP regimen as the first-line treatment. After two cycles of EP chemotherapy, the patient’s creatinine levels continued to rise, and gastrointestinal side effects were significant. The patient’s symptoms, tumor markers, and imaging findings also improved. The ECOG performance status score increased from 0 to 2. All these parameters indicated treatment failure of the first-line EP chemotherapy. After a multidisciplinary team (MDT) discussion and considering the patient’s condition, second-line chemotherapy with irinotecan combined with carboplatin was administered according to the 2022 NCCN guidelines. However, after second-line chemotherapy, re-evaluation showed that NSE and related tumor markers were still elevated, imaging suggested further progression, and the patient experienced significant side effects during chemotherapy, indicating failure of second-line irinotecan combined with carboplatin chemotherapy.

There is no standard third-line therapy for NEPC, and the patient’s tumor continues to progress rapidly. We performed a literature review and found that tyrosine kinase inhibitors (TKIs), such as anlotinib, combined with PD-1 inhibitors, have shown good therapeutic effects and safety in SCLC in recent years. Zhang et al. retrospectively analyzed patients with recurrent SCLC who received anlotinib combined with a PD-1 inhibitor as ≥ second-line treatment and found that the progression-free survival (PFS) of patients receiving combination therapy was significantly longer than that of patients receiving PD-1 inhibitors alone (n = 14, 5.0 months vs. 3.0 months; P = 0.005), suggesting that anlotinib combined with PD-1 inhibitors may have good efficacy and manageable toxicity in SCLC (10). Zhang et al. described a patient aged >70 years with extensive-stage SCLC who achieved good survival (median overall survival of 13 months) and safety with third-line pembrolizumab combined with anlotinib therapy after the failure of standard treatment (11). Hao et al. retrospectively analyzed 36 SCLC patients who had received at least one systemic chemotherapy regimen and found that the objective response rate (ORR) was 27.8% (95% CI: 14.2%–45.2%) and the disease control rate (DCR) was 80.6% (95% CI: 64.0%–91.8%), indicating that anlotinib combined with a PD-1 inhibitor has significant efficacy and safety for previously treated SCLC patients (12).

Therefore, after MDT discussion and after thorough discussion and informed consent from the patient, we decided to treat the patient with a combination of anlotinib and tislelizumab as an innovative attempt. Subsequently, the patient received treatment every three weeks for a cycle, showing good tolerance and without significant side effects. NSE and related tumor markers dramatically decreased, tumor reduction was significant on imaging, and the treatment efficacy was remarkable. The ECOG performance status score recovered to 1, and the patient’s physical condition improved. The patient has survived for more than two years, having received over 25 treatment cycles.

Anlotinib is a multi-target tyrosine kinase inhibitor (TKI). It can inhibit various targets including vascular endothelial growth factor receptors (VEGFR), platelet-derived growth factor receptors (PDGFR), fibroblast growth factor receptors (FGFR), and c-Kit (13). It inhibits tumor cell proliferation, invasion, and metastasis and reduces tumor blood supply by inhibiting tumor angiogenesis and growth factor signaling pathways. Compared with other TKIs, anlotinib has better anti-angiogenic activity and higher selectivity, with a significantly lower incidence of grade 3 or higher adverse effects (14, 15).

Tislelizumab (BGB-A317) is a novel anti-PD-1 monoclonal antibody that blocks the PD-1/PD-L1 signaling pathway, relieves the immune suppression of T cells, and enhances the anti-tumor immune response. Compared to traditional anti-PD-1 antibodies, its Fc region has been structurally optimized, resulting in stronger antitumor activity compared to nivolumab and pembrolizumab. In the 2023 WCLC, tislelizumab combined with chemotherapy demonstrated significant survival benefits in extensive-stage small cell lung cancer (ES-SCLC) (RATIONALE 312, NCT04005716), with OS extending to 15.5 months (versus 13.5 months in the control group, HR: 0.75, 95% CI: 0.61–0.92, P = 0.0035), and the 1-year PFS rate was four times that of the control group (20.7% vs. 4.5%) (16).

Studies on the efficacy of immunotherapy for NEPC are limited. Although prostate cancer is typically considered a “cold tumor,” the immune microenvironment of primary NEPC remains largely unknown. Theoretically, anti-angiogenic agents can inhibit tumor growth and metastasis by disrupting the tumor blood supply, resulting in a lack of oxygen and nutrients. However, after anti-angiogenesis therapy, a complex local balance exists between pro-angiogenic factors, anti-angiogenic factors, and vascular normalization (17). During tumor vascular normalization, the function of immunosuppressive cells such as regulatory T cells (Tregs), tumor-associated macrophages (TAMs), and myeloid-derived suppressor cells (MDSCs) may be weakened, thereby improving the immune microenvironment of the tumor (17, 18). Furthermore, these suppressive cells not only affect tumor immune responses through immune suppression but also stimulate tumor angiogenesis by increasing the expression of pro-angiogenic factors in the extracellular matrix. There seems to be a reciprocal regulation between the immune response and vascular normalization (19). The combination of immune checkpoint inhibitors (ICIs) and anti-angiogenic agents may have potential synergistic anti-tumor effects.

Du et al. conducted a retrospective real-world study on 25 patients with mCRPC who received PD-1 inhibitors combined with anlotinib after progression to standard treatment. The study found that six patients (24.0%) exhibited a prostate-specific antigen (PSA) response and 11 patients (44.0%) experienced a decrease in PSA levels (20). Additionally, the study found that mCRPC patients with DNA damage repair (DDR) and homologous recombination repair (HRR) defects had relatively longer PSA progression-free survival (PSA-PFS; 2.5 months vs. 1.2 months, P = 0.027; 3.3 months vs. 1.2 months, P = 0.017) (20). However, Du’s report did not include cases of NEPC, and our case suggests that patients with NEPC may also benefit from anlotinib combined with PD-1 inhibitor treatment.

Unfortunately, during the COVID-19 pandemic, the patient was hospitalized twice for severe COVID-19 infection, leading to interruption of anlotinib combined with tislelizumab treatment. After recovering from COVID-19, the MDT discussion decided to continue the anlotinib and tislelizumab treatment cycles. In November 2023, the patient experienced a stroke, which led to another interruption in treatment. The MDT assessed that stroke was less likely to be related to anlotinib and tislelizumab treatment. After recovering from stroke, the patient resumed anlotinib and tislelizumab treatment again. The patient is currently undergoing treatment, but these attacks have impeded the patient’s physical condition. Currently his ECOG performance status score is 3. These concomitant diseases may significantly reduce a patient’s expected OS.

In brief, this elderly patient was diagnosed with metastatic primary small cell NEPC. After treatment failure of the first- and second-line chemotherapy according to the guidelines, the patient was treated with anlotinib and tislelizumab. This combination led to a significant reduction in NSE and other related tumor markers, and imaging revealed notable tumor shrinkage. Progression-free survival (rPFS) exceeded 22 months. Although overall survival (OS) data are not yet available, the patient survived for more than two years after diagnosis. This case provides valuable insights into the future management of similar patients.

## 4 Conclusion

To our knowledge, this is the first report of the use of anlotinib combined with tislelizumab for the treatment of primary small cell NEPC, achieving good efficacy and safety in this patient. This case suggests that for patients with primary small cell NEPC, anlotinib-targeted therapy combined with PD-1 inhibitor immunotherapy may bring significant benefits. This case indicates that in NEPC patients, after the failure of standard treatments, anlotinib combined with tislelizumab can be considered as a preferred treatment option. Additionally, we recommend exploring anlotinib combined with tislelizumab as a first-line treatment choice in patients with NEPC. More clinical trials and real-world data are needed to support the application of anlotinib in combination with PD-1 inhibitors in primary NEPC.

## Data Availability

The original contributions presented in the study are included in the article/**Supplementary Material**. Further inquiries can be directed to the corresponding authors.

## Supplementary material

Supplementary Figure 1 expression of PD-L1 in this patient. Results of PD-L1 immunohistochemistry (IHC) in this patient. **(A)** Hematoxylin and eosin (H&E) staining; **(B)** PD-L1 negative control; **(C)** PD-L1 positive control; **(D)** PD-L1 IHC result of the patient, showing negativity (-).

Supplementary Figure 2 Non-contrast CT scan of the kidneys, ureters, and bladder (March 2022). showed tumor invaded to both the ureteral orifice.

Supplementary Video 1 Non-contrast CT scan of the kidneys, ureters, and bladder of this patient (March 2022).

Supplementary Video 2 Non-contrast CT scan of the kidneys, ureters, and bladder of this patient (April 2022).

## References

[1] Aggarwal R Huang J Alumkal JJ Zhang L Feng FY Thomas GV. Clinical and genomic characterization of treatment-emergent small-cell neuroendocrine prostate cancer: A multi-institutional prospective study. J Clin Oncol Off J Am Soc Clin Oncol. (2018) 36:2492–503. doi: 10.1200/JCO.2017.77.6880 PMC6366813 29985747

[2] Lee AR Gan Y Xie N Ramnarine VR Lovnicki JM Dong X. Alternative RNA splicing of the GIT1 gene is associated with neuroendocrine prostate cancer. Cancer Sci. (2019) 110:245–55. doi: 10.1111/cas.13869 PMC6317919 30417466

[3] Aggarwal R Zhang T Small EJ Armstrong AJ. Neuroendocrine prostate cancer: subtypes, biology, and clinical outcomes. J Natl Compr Canc Netw. (2014) 12:719–26. doi: 10.6004/jnccn.2014.0073 24812138

[4] Zhang Y Ouyang W Sun G Ding B Yan L Wang Z. Pure small cell carcinoma of prostate: A report of 8 cases. Urol Int. (2018) 101:263–8. doi: 10.1159/000493160 30269133

[5] Wee CE Costello BA Orme JJ Quevedo JF Pagliaro LC. Chemotherapy with atezolizumab for small cell or neuroendocrine carcinoma of the prostate: A single institution experience. Prostate. (2021) 81:938–43. doi: 10.1002/pros.24189 34254332

[6] Deorah S Rao MB Raman R Gaitonde K Donovan JF. Survival of patients with small cell carcinoma of the prostate during 1973-2003: a population-based study. BJU Int. (2012) 109:824–30. doi: 10.1111/j.1464-410X.2011.10523.x 21883857

[7] Marcus DM Goodman M Jani AB Osunkoya AO Rossi PJ. A comprehensive review of incidence and survival in patients with rare histological variants of prostate cancer in the United States from 1973 to 2008. Prostate Cancer Prostatic Dis. (2012) 15:283–8. doi: 10.1038/pcan.2012.4 22349984

[8] Faivre-Finn C Snee M Ashcroft L Appel W Barlesi F Bhatnagar A. Concurrent once-daily versus twice-daily chemoradiotherapy in patients with limited-stage small-cell lung cancer (CONVERT): an open-label, phase 3, randomised, superiority trial. Lancet Oncol. (2017) 18:1116–25. doi: 10.1016/S1470-2045(17)30318-2 PMC5555437 28642008

[9] Goldman JW Dvorkin M Chen Y Reinmuth N Hotta K Trukhin D. Durvalumab, with or without tremelimumab, plus platinum-etoposide versus platinum-etoposide alone in first-line treatment of extensive-stage small-cell lung cancer (CASPIAN): updated results from a randomised, controlled, open-label, phase 3 trial. Lancet Oncol. (2021) 22:51–65. doi: 10.1016/S1470-2045(20)30539-8 33285097

[10] Zhang X Zeng L Li Y Xu Q Yang H Lizaso A. Anlotinib combined with PD-1 blockade for the treatment of lung cancer: a real-world retrospective study in China. Cancer Immunol Immunother CII. (2021) 70:2517–28. doi: 10.1007/s00262-021-02869-9 PMC10991983 33566148

[11] Zhang Z Li Y Dong Y Li J Zhang B Zhang C. Successful treatment of a patient with multiple-line relapsed extensive-stage small-cell lung cancer receiving penpulimab combined with anlotinib: A case report. Front Oncol. (2022) 12:846597. doi: 10.3389/fonc.2022.846597 35321433 PMC8937034

[12] Hao YY Qiao YP Cheng JD. Clinical activity and safety of anlotinib combined with PD-1 blockades for patients with previously treated small cell lung cancer. Int J Gen Med. (2021) 14:10483–93. doi: 10.2147/IJGM.S337316 PMC8722563 35002304

[13] Shen G Zheng F Ren D Du F Dong Q Wang Z. Anlotinib: a novel multi-targeting tyrosine kinase inhibitor in clinical development. J Hematol OncolJ Hematol Oncol. (2018) 11:120. doi: 10.1186/s13045-018-0664-7 30231931 PMC6146601

[14] Xie C Wan X Quan H Zheng M Fu L Li Y. Preclinical characterization of anlotinib, a highly potent and selective vascular endothelial growth factor receptor-2 inhibitor. Cancer Sci. (2018) 109:1207–19. doi: 10.1111/cas.13536 PMC5891194 29446853

[15] Cao JZ Wu W Pan JF Wang HW Jiang JH Ma Q. Case report: anlotinib combined with sintilimab as third-line treatment in a metastatic urothelial bladder carcinoma patient with FGFR3 mutation. Front Oncol. (2021) 11:643413. doi: 10.3389/fonc.2021.643413 34109111 PMC8180869

[16] Cheng Y Fan Y Zhao Y Huang D Li X Zhang P. OA01.06 first-line chemotherapy with or without tislelizumab for extensive-stage small cell lung cancer: RATIONALE-312 phase 3 study. J Thorac Oncol. (2023) 18:S46. doi: 10.1016/j.jtho.2023.09.027

[17] Kazerounian S Lawler J. Integration of pro- and anti-angiogenic signals by endothelial cells. J Cell Commun Signal. (2018) 12:171–9. doi: 10.1007/s12079-017-0433-3 PMC5842194 29264709

[18] Tian L Goldstein A Wang H Ching Lo H Sun Kim I Welte T. Mutual regulation of tumour vessel normalization and immunostimulatory reprogramming. Nature. (2017) 544:250–4. doi: 10.1038/nature21724 PMC5788037 28371798

[19] Fukumura D Kloepper J Amoozgar Z Duda DG Jain RK. Enhancing cancer immunotherapy using antiangiogenics: opportunities and challenges. Nat Rev Clin Oncol. (2018) 15:325–40. doi: 10.1038/nrclinonc.2018.29 PMC5921900 29508855

[20] Du XX Dong YH Zhu HJ Fei XC Gong YM Xia BB. PD-1 inhibitor plus anlotinib for metastatic castration-resistant prostate cancer: a real-world study. Asian J Androl. (2023) 25:179–83. doi: 10.4103/aja2022102 PMC10069691 36537376

