# Supplementary figures and images for: Anlotinib combined with tislelizumab in the treatment of primary small cell neuroendocrine carcinoma of the prostate: a case report and literature review
Source: Front Immunol. 2024 Dec 23;15:1510069. doi: 10.3389/fimmu.2024.1510069 (PMC11701218; doi:10.3389/fimmu.2024.1510069)

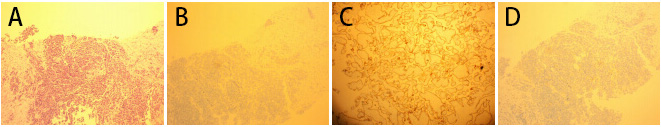

Supplement: Supplementary Figure 1 — expression of PD-L1 in this patient. Results of PD-L1 immunohistochemistry (IHC) in this patient. (A) Hematoxylin and eosin (H&E) staining; (B) PD-L1 negative control; (C) PD-L1 positive control; (D) PD-L1 IHC result of the patient, showing negativity (-). [file Image1.jpeg]

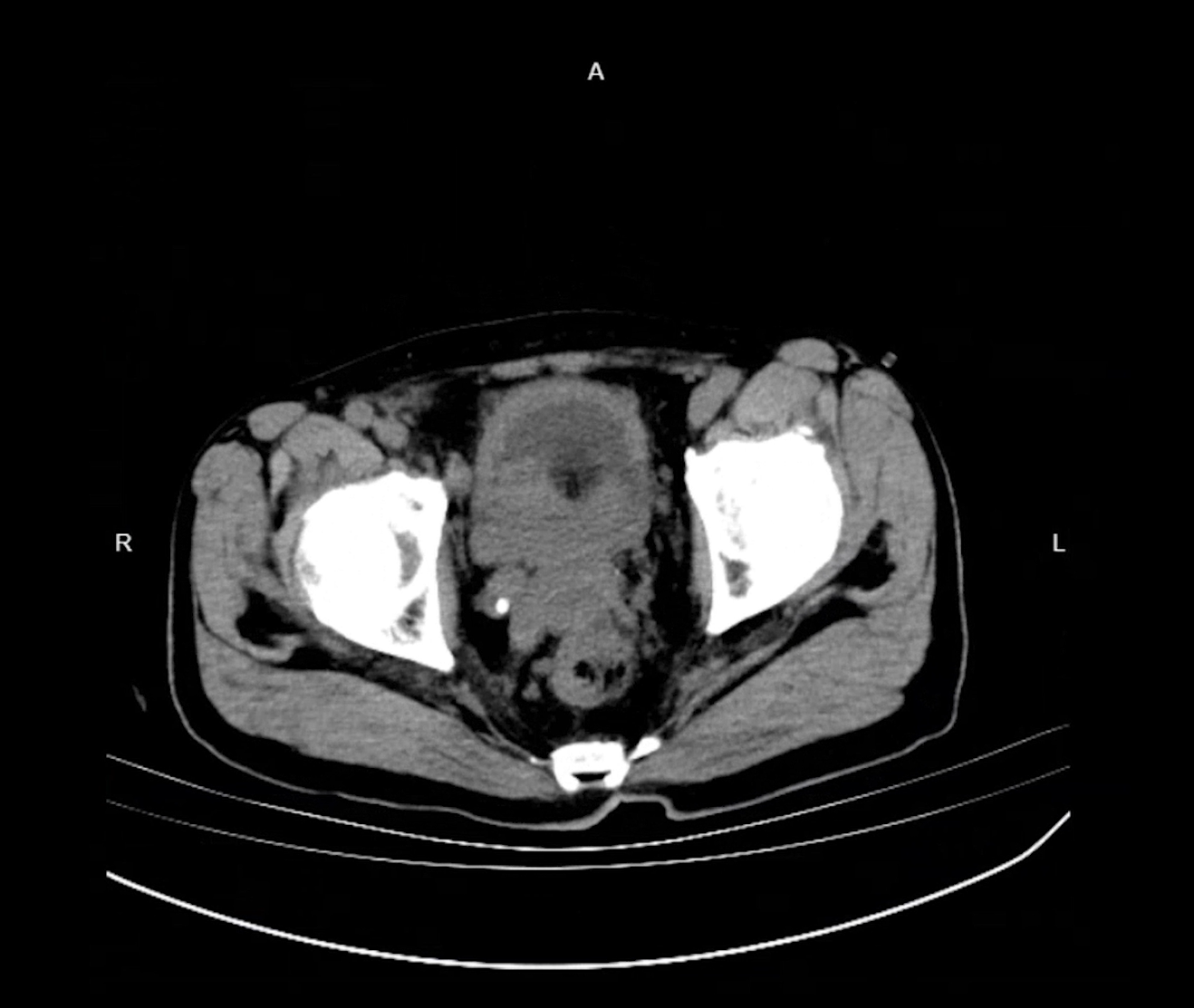

Supplement: Supplementary Figure 2 — Non-contrast CT scan of the kidneys, ureters, and bladder (March 2022). showed tumor invaded to both the ureteral orifice. [file Image2.png]
